# The tropics should not become the world's plastic pollution problem

**DOI:** 10.1177/27538931231165273

**Published:** 2023-04-05

**Authors:** Tony R Walker

**Affiliations:** School for Resource and Environmental Studies, Dalhousie University, Halifax, Canada

**Keywords:** Single-use plastics, plastic waste, plastic pollution, plastic policies, sustainability, plastics treaty

## Abstract

Unsustainable plastic production, use and mismanagement have resulted in increased plastic pollution in the environment threatening sustainability, especially in the tropics. Countries in the tropics have been disproportionally impacted by plastic pollution due to imports of plastic waste from developed countries, or because tropical Small Island Developing States have become overwhelmed by single-use plastics used widely in the tourism sector. However, plastic pollution is pervasive and is not just limited to the tropics. Plastic pollution has resulted in widespread environmental, economic and social impacts globally. Most plastics are derived from fossil fuels which contribute to climate change via greenhouse gas emissions, and plastic pollution also harms wildlife threatening biodiversity, thus placing enormous pressure on earth’s limited resources. Although downstream strategies to curb plastic pollution exist, they are infective in the face of increased upstream plastic production. Therefore, the international community has recognized that a more holistic approach is required to reduce plastic pollution. Current plastic production and waste generation are still outpacing existing plastic reduction regulations. This viewpoint shows why unsustainable global plastic production has resulted in increased global plastic pollution, including in the tropics, but also highlights how ambitious plastic pollution reduction policies can help transition towards a more sustainable plastics future.

## Introduction

Plastic pollution has been a growing concern globally for decades (Walker, 2018a). Unsustainable plastic production, use and mismanagement have resulted in increased plastic pollution in the environment threatening sustainability, especially in the tropics (Costa et al., 2011; Possatto et al., 2011). In 2019, global plastic production reached 368 million metric tons (Mt) (Plastics Europe, 2020), but is estimated to double in the next two decades (Lebreton and Andrady, 2019). As many consumer plastics are designed and intended for single-use, this has resulted in increasing quantities of plastic waste and widespread plastic pollution (McGuinty and Walker, 2023). Borrelle et al. (2020) estimated that an astonishing 19–23 Mt of plastic waste generated across the globe in 2016 entered aquatic ecosystems. This is predicted to reach up to 53 Mt annually by 2030, which is just 7 years away. Globally, since 1950, an estimated 9200 Mt and upwards of 6900 Mt (∼75%) of plastic has ended up in landfills or worse, polluting the environment (Geyer et al., 2017). Alarmingly, little of this plastic waste is recycled. For example, in 2019, only 9% of plastic waste was recycled, while 19% was incinerated and 22% was mismanaged, globally (OECD, 2022).

Plastic waste mismanagement is even worse in tropical countries (OECD, 2022). Countries in the tropics have been disproportionally impacted by plastic pollution due to imports of plastic waste from developed countries (Liu et al., 2018; Walker, 2018b). For example, developed countries have been exporting their plastic waste to developing countries in the tropics for decades. This trend has been increasing in recent years. After China closed its doors to plastic waste imports in 2018, developed countries began exporting plastic waste to other developing countries, including countries in the tropics (e.g. Brooks et al., 2018; Liu et al., 2018; Law et al., 2020). However, many of these importing countries lack adequate waste management facilities, which have led to excessive open dumping or burning of plastic waste, including waste-to-energy incineration (Jambeck et al., 2015). Burning plastic waste for energy releases greenhouse gases, contributes to accelerating climate change and produces toxic atmospheric pollutants impacting local communities and marine ecosystems (DeWeerdt, 2022).

Whilst many developing countries in the tropics lack formal waste management infrastructure, informal waste pickers, provide a valuable way to recover and recycle plastics, which otherwise could leak into the environment or be incinerated (Cook et al., 2022). Unfortunately, waste pickers often lack personal safety equipment, because of unsafe working conditions in these dumpsites. Thus, plastic waste management, especially in developing countries can lead to increased health risks and exposure to toxic chemicals related to unhealthy waste handling for waste workers (Cook et al., 2022; Kazemi Moghaddam et al., 2023). Another reason why some countries in the tropics have been disproportionally impacted by plastic pollution is because Small Island Developing States (SIDSs) have become overwhelmed by the consumption of single-use plastics used extensively in the tourism sector (Ambrose et al., 2019). For example, plastic pollution is a particular problem for Caribbean countries, as they are major contributors of plastic marine pollution, but are also heavily dependent on maintaining the beauty of the Caribbean Sea, which is a major attraction for the tourism industry which contributes 15.5% of the Caribbean GDP (Clayton et al., 2021). Although countries in the tropics have suffered disproportionately from the impacts of plastic pollution, plastic pollution is pervasive and is not just limited to the tropics.

Plastic pollution has therefore resulted in widespread environmental, economic and social impacts not just in the tropics, but globally (Beaumont et al., 2019). Because plastic pollution recognizes no international boundaries it has become a global persistent pollution problem requiring many solutions to reduce sources of plastic pollution (i.e. leakage) at local, national and international levels (Walker, 2018a; Xanthos and Walker, 2017; Schnurr et al., 2018; ASEAN Framework of Action on Marine Debris, 2019). Recognizing the scale of the problem, there have been many initiatives at different levels to reduce plastic waste, including mechanisms and policies, communication, and plastic collection and treatment, with the extensive participation of international organizations, governmental and non-governmental organizations, as well as corporations in the private sector (Prata et al., 2019; Adam et al., 2020; Bezerra et al., 2021; Clayton et al., 2021).

Many tropical countries in Southeast Asia, including some of the top countries contributing to plastic leakage, have been recognized as a hot spot for plastic pollution, due to rapid urbanization, rising middle class and inadequate infrastructure for waste management (Jambeck et al., 2015). The current plastics economy, especially single-use plastics is generally a linear cycle of take, make and waste (Borrelle et al., 2020). According to the World Bank Group, an estimated 95% of single-use plastic packaging's value – US$80 to US$120 billion a year – is lost to the economy when single-use plastic items are discarded rather than recovered and recycled (Van Trotsenburg and Hoi, 2022). Countries across Southeast Asia have been preparing regional and national action plans and circular economy road maps to prioritize plastic pollution monitoring and associated plastic reduction policies and investments in key sectors and locations (Van Trotsenburg and Hoi, 2022). Recognizing the transboundary nature of marine plastics, the Association of Southeast Asian Nations (ASEAN) released the Bangkok Declaration on Combating Marine Plastics in 2019 (ASEAN Framework of Action on Marine Debris, 2019; Van Trotsenburg and Hoi, 2022). In the ASEAN region, marine debris is a transboundary issue requiring integrated and robust regional and national action plans to address marine debris. In the ASEAN Framework of Action on Marine Debris, four priority objectives and priority areas include: (i) policy support and planning; (ii) research, innovation and capacity building; (iii) public awareness, education and outreach; and (iv) private sector engagement (ASEAN Framework of Action on Marine Debris, 2019; ASEAN, 2021).

Many international organizations have called for standardized and regular monitoring, and management of plastic pollution data (Walker et al., 2021). Recent marine plastic debris monitoring frameworks from international organizations such as the UNEP, ASEAN, GESAMP, G7 Oceans Plastics Charter, G20 Nations, and other national and regional bodies, recognize a need to understand the long-term changes in marine plastics to successfully develop and implement mitigation strategies (ASEAN, 2021; Walker et al., 2021). Enforcement of existing international, regional or national regulations is lacking and undermines the ability of the international community to implement solutions to reduce plastic pollution (Walker et al., 2021; Walker, 2021a).

Countries across the ASEAN region are using innovative methods to measure and monitor plastic pollution on land, in rivers and in the marine environment (e.g. drone monitoring in Cambodia and baseline monitoring assessments of plastic pollution in Indonesia and Vietnam (Walker et al., 2021; Van Trotsenburg and Hoi, 2022). Across the tropics and globally, successful examples of market-based instruments and legislation are being implemented, such as plastic bag taxes, bans on certain plastic products and bottle deposit refund schemes (Xanthos and Walker, 2017; Schnurr et al., 2018; Adam et al., 2020; Bezerra et al., 2021; Clayton et al., 2021). Policies such as extended producer responsibility (EPR) are being explored across the tropics and globally, to help ensure that corporations pushing plastic products on the market are required to pay for their collection, sorting and recycling after use and end-of-life (Diggle and Walker, 2020; Diggle and Walker, 2022). Thus, there are many policies available for governments in the tropics to reduce single-use plastics use and waste and improve the recycling of plastics (Table 1).

**Table 1.** Policies to minimize single-use plastics (modified from UNEP, 2018).

Single-use plastic reduction policies	Policy implementation strategies	Policy examples	Policy outcomes
Voluntary reduction	Requires agreements of all stakeholders (government, consumers and industry) Voluntary consumer choices emerge with increased awareness	Voluntary agreements between all stakeholders Agreements between industry and governments (e.g. extended producer responsibility, EPR) Reusable alternatives to single-use plastics are widely available	Voluntary actions allow all stakeholders to adopt single-use plastic reduction initiatives (e.g. switching to reusables or sustainable plastic alternatives)
Increase public education and awareness	Interventions required to change to pro-environmental consumer behaviour	Environmental education in schools Public social media education campaigns	Fosters pro-environmental consumer behaviour
Types of policies	Legislative or regulatory policies Market-based or economic policies Combination of legislative and market-based policies	Policies vary widely according to jurisdiction or waste management and recycling infrastructure available (e.g. legislative bans, taxes, or levies on single-use plastics, EPR and independent corporate sustainability actions)	Reduced use and disposal of single-use plastics by consumers and industry Tax revenues from market-based policies can be used in government waste reduction initiatives and education

**Figure 1.**
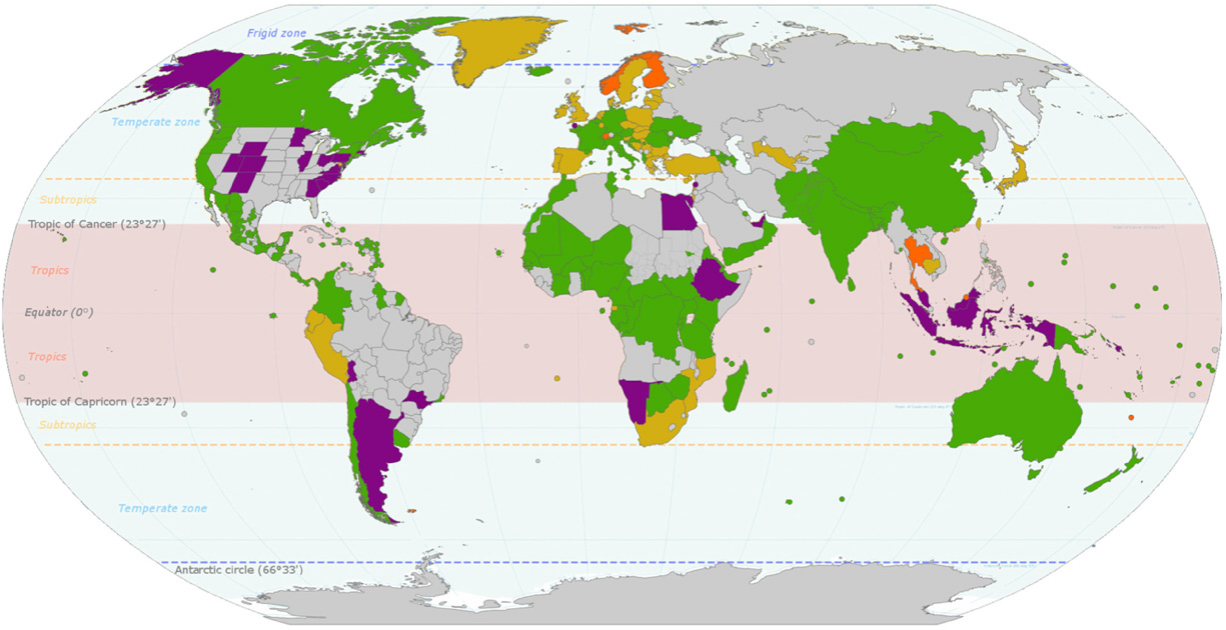
Global plastic bag policies with tropical countries are indicated within the pink band. Green, indicates an outright plastic bag ban; yellow, indicates a charge on some plastic bags; orange, indicates a voluntary charge; purple, indicates a partial plastic bag charge or ban at municipal or regional levels (adapted from Elekhh – Own work, CC BY-SA 3.0, https://commons.wikimedia.org/w/index.php?curid = 32400659).

Policies include legislative bans on certain types of plastics. This has historically been focused on plastic shopping bags (Figure 1), but recently legislative bans have been expanded to include bans on microbeads and other types of single-use plastics (e.g. food packaging and utensils) (Xanthos and Walker, 2017; Schnurr et al., 2018). Other policies to reduce single-use plastics include taxes and levies (both are charges) and are designed to change human behaviour and dissuade the use of certain types of plastics (i.e. problem or harmful plastics found in the environment. Revenues from taxes or levies have been used in other jurisdictions to fund green initiatives, such as education, and fund recycling programs. Finally, another policy for governments to consider implementing is EPR. EPR strategies leverage corporate resources to reduce single-use plastic waste generated by consumers. Implementation of EPR strategies allows local jurisdictions to gain greater control of their waste management (Diggle and Walker, 2020; Diggle and Walker, 2022).

Whilst policy instruments have had varying degrees of success around the world to reduce sources of marine plastic pollution, top-down approaches may sometimes be difficult to implement (Clayton et al., 2021; Bezerra et al., 2021), can be ill-conceived by governments if consumers have not been consulted (Nwafor and Walker, 2020) and may be unpopular with the plastics industry, manufacturers, retailers and consumers lacking environmental awareness (Schnurr et al., 2018). However, bottom-up approaches have been successful at reducing the use of single-use plastics and ultimately sources of plastic pollution. Bottom-up approaches include voluntary reduction strategies by consumers and businesses alike as well as raising public awareness about the environmental threats posed by plastic pollution (Varkey et al., 2021).

Voluntary reduction strategies by consumers like the use of reusable water bottles, bags and straws has become increasingly popular following popular media campaigns such as the ‘*Blue Planet II*’ documentary by the BBC and the National Geographic slogan ‘*Planet or Plastic*’ to raise awareness about the global plastic pollution crisis (Schnurr et al., 2018). Industrial voluntary reduction strategies include business recognition programs and certifications for plastic waste reduction, in addition to the gamification and incentivization for consumer rewards to enhance reuse (Varkey et al., 2021). Public education and raising awareness can take many forms, but successful awareness campaigns include voluntary beach clean-ups by citizen scientists. Beach clean-ups by citizen scientists can help remove stranded beach debris, although removal rates of plastic pollution pale compared to plastic pollution inputs into the world's oceans. However, one of the most impactful outcomes of empowering citizen scientists is that it helps compile useful plastic debris characterization data to help inform government policy and helps trigger behaviour change and pro-environmental actions (Ambrose et al., 2019; Walker et al., 2021).

The 10-step roadmap developed by UNEP (2018) below, helps guide governments to implement effective policies to reduce plastic pollution (Table 2).

**Table 2.** Roadmap to implement effective policies to reduce plastic pollution (modified from UNEP, 2018).


Assess baseline conditions	Assessing baseline conditions provides policymakers with evidence of plastic categories commonly found in the environment and helps determine what are the most problematic plastics requiring government action (UNEP, 2018; Ambrose et al., 2019; Walker et al., 2021).
Evaluate appropriateness of potential policies	After baseline surveys evaluate appropriate policies (e.g. levies or bans on plastic grocery bags) to address specific issues identified (Walker et al., 2021).
Assess sustainability of policies	Study the sustainability of the short-listed policies by considering all sectors (e.g. food and/or other retail) and all segments of the population. Although the environmental benefits of a ban might be positive, the social impacts on some of the population might be unsustainable, making bans undesirable. For example, in many African countries, the effectiveness of outright bans on plastic bags has not been proven due to a lack of enforcement and a lack of suitable sustainable alternatives to plastic bags (Adam et al., 2020; Nwafor and Walker, 2020).
Stakeholder engagement	Acceptance by all stakeholders is crucial and can be achieved through calls for public input to policy discussions, and wide-reaching awareness campaigns, although opposition from industry should be anticipated, especially when using regulatory instruments (Walker, 2021b).
Raise awareness	Consumer acceptance is more likely if consumers are aware of the social, environmental and economic impacts of mismanaged single-use plastics which can be communicated via education programs in schools, extensive multi-media awareness-raising campaigns, development and distribution of information material and demonstrating and/or distributing reusable alternatives. Campaigns should have a clear and simple message, relevant for a wide range of stakeholders (UNEP, 2018).
Support uptake of sustainable alternatives	Before implementing bans, governments should ensure the availability of appropriate sustainable alternatives (Nwafor and Walker, 2020). If affordable alternatives are lacking, bans may end up negatively impacting the poorest members of society (UNEP, 2018). A common alternative to replacing plastic bags is paper. Although paper degrades naturally in the environment and has no pollution legacy, unlike plastic, they require more energy to produce.
Provide incentives to industry	Governments may incur lobbying by the plastic industry and packaging importers if regulating the production and consumption of single-use plastics (Walker, 2021c). To limit resistance and gain support, governments should offer incentives to industry before new legislation is implemented to guarantee sufficient time for plastic manufacturers, distributors and retailers to adapt to the new stipulations (Clayton et al., 2021; Bezerra et al., 2021).
Use revenues to support environmental programs	To maximize public benefits, revenues from levies could be used to support specific environmental projects, boost the local recycling industry and create job opportunities in plastic recycling (Xanthos and Walker, 2017). To maximize public support, it is crucial to communicate how revenues will be used (UNEP, 2018).
Policy enforcement	To guarantee good governance, enforcement and monitoring of policies, it is important to clearly define roles and responsibilities between local, national and sub-national governments and organizations to ensure compliance with the policy (UNEP, 2018).
Continue to monitor plastic pollution and adjust policies	It is important to monitor the progress and effectiveness of introduced policies and adjust accordingly. Progress could be monitored through audits, surveys, impact assessments and focus-group interviews. To gather data on effectiveness, governments should develop a monitoring database to allow for comparability across years to estimate the reduction in plastic pollution (Walker et al., 2021).

Although these downstream strategies to curb plastic pollution exist, they are mostly consumer focused and are ineffective in the face of increased upstream plastic production. Consequently, the international community has now recognized that a more holistic approach is required to reduce plastic pollution. In March 2022, the United Nations Environment Assembly adopted a resolution to curb plastic pollution with a legally binding global plastics treaty. If the plastics treaty is to be successful, it must be legally binding and adopted by all United Nations member states (Ammendolia and Walker, 2022).

Countries in the tropics have the most to lose from the devastating impacts of plastic pollution if the Plastics Treaty negotiations and implementation are unsuccessful. Recognizing this, many tropical countries are already members of the High Ambition Coalition (HAC) and have made ambitious commitments to end plastic pollution by 2040. As of 25 January 2023, the HAC has 51 members, including many tropical countries: Rwanda, Peru, Senegal, Georgia, the Republic of Korea, Costa Rica, Ecuador, the Dominican Republic, Ghana, Cabo Verde, Burkina Faso, Colombia, Mali, Montenegro, Cook Islands, Mexico, Guinea, Antigua and Barbuda, and the Maldives (High Ambition Coalition, 2023). However, the strong commitments made by members of the HAC, such as global standards, bans and restrictions on virgin plastic production (Bergmann et al., 2022; Dey et al., 2022), may be undermined by countries like as the United States who are seeking to form another, less ambitious, coalition of countries to drive negotiations on the global plastics treaty (Ammendolia and Walker, 2022; Walker, 2022). The United States wants to model the treaty on voluntary efforts like the 2015 Paris climate agreement, rather than provide new internationally legally binding rules favoured by HAC member states. The weaker model proposed by the United States would lack strict global measures such as banning or curbing plastic production, which has been strongly opposed by the powerful plastics industry (Walker, 2021b). The plastics treaty must be ambitious and include agreements by all United Nations member states including global standards, bans and restrictions on virgin plastic production.

## Conclusions

Countries in the tropics have been and continue to be disproportionally impacted by plastic pollution due to imports of plastic waste from developed countries. These imports are often illegal or comprise of low-quality plastic which increases the likelihood of these plastics being incinerated, landfilled or leaking into the environment. Unsurprisingly, plastic pollution continues to be pervasive in countries in the tropics. Although there are some policies and strategies to curb plastic pollution, they are implemented only at national or regional levels and are inconsistently enforced. Plastic pollution reduction policies are also ineffective in the face of increased plastic production, even when properly implemented and enforced. Thus, the international community has recognized that a more holistic approach is required to reduce plastic pollution. This holistic approach must include caps on virgin plastic production if we are truly committed to curb plastic pollution. Currently, negotiations are underway to curb plastic pollution with a legally binding global plastics treaty. As countries in the tropics have the most to lose from the negative impacts of plastic pollution, many tropical countries have already made ambitious commitments to end plastic pollution by 2040.
